# Mechanisms and Perspectives of Sodium-Glucose Co-transporter 2 Inhibitors in Heart Failure

**DOI:** 10.3389/fcvm.2021.636152

**Published:** 2021-02-10

**Authors:** Qingchun Zeng, Qing Zhou, Weitao Liu, Yutong Wang, Xingbo Xu, Dingli Xu

**Affiliations:** ^1^State Key Laboratory of Organ Failure Research, Department of Cardiology, Nanfang Hospital, Southern Medical University, Guangzhou, China; ^2^Guangdong Provincial Key Laboratory of Shock and Microcirculation, Southern Medical University, Guangzhou, China; ^3^Bioland Laboratory (Guangzhou Regenerative Medicine and Health Guangdong Laboratory), Guangzhou, China; ^4^Department of Cardiology, Xiangyang Central Hospital, Affiliated Hospital of Hubei University of Arts and Science, Xiangyang, China; ^5^Department of Cardiology and Pneumology, University Medical Center of Göttingen, Georg-August-University, Göttingen, Germany

**Keywords:** sodium-glucose co-transporter 2 inhibitors, diabetes mellitus, heart failure, heart failure with preserved ejection fraction, insulin resistance

## Abstract

Heart failure (HF) is a common complication or late-stage manifestation of various heart diseases. Numerous risk factors and underlying causes may contribute to the occurrence and progression of HF. The pathophysiological mechanisms of HF are very complicated. Despite accumulating advances in treatment for HF during recent decades, it remains an intractable clinical syndrome with poor outcomes, significantly reducing the quality of life and expectancy of patients, and imposing a heavy economic burden on society and families. Although initially classified as antidiabetic agents, sodium-glucose co-transporter 2 (SGLT2) inhibitors have demonstrated reduced the prevalence of hospitalization for HF, cardiovascular death, and all-cause death in several large-scale randomized controlled clinical trials. These beneficial effects of SGLT-2 inhibitors can be attributed to multiple hemodynamic, inflammatory and metabolic mechanisms, not only reducing the serum glucose level. SGLT2 inhibitors have been used increasingly in treatment for patients with HF with reduced ejection fraction due to their surprising performance in improving the prognosis. In addition, their roles and mechanisms in patients with HF with preserved ejection fraction or acute HF have also attracted attention. In this review article, we discuss the possible mechanisms and applications of SGLT2 inhibitors in HF.

## Introduction

Heart failure (HF) has become an increasingly serious public-health problem worldwide (1). HF plagues not only high-income countries such as the USA and European countries, but also low- and middle-income countries (2). HF seriously affects the quality of life (QoL) and leads to significantly shortened life expectancy. Also, the HF-related economic burden is significant and will increase as populations age (1). Despite considerable progress in drug treatment and device therapies as well as widespread implementation of preventive measures, the prevalence of rehospitalization and mortality of HF patients remains unacceptably high (3).

Type 2 diabetes mellitus (T2DM) is also prevalent worldwide. Nearly 10% of the adult population worldwide (~382 million) suffers from T2DM. More importantly, this number is increasing and has been projected to reach 592 million by 2035 (4).

There is a close relationship between T2DM and HF. Cardiovascular (CV) diseases remain the leading cause of disability and death among patients with T2DM. About two-thirds of people with T2DM eventually die from CV diseases, including atherosclerosis, myocardial infarction (MI), HF, or stroke (5). Conversely, T2DM is an important predictor of new-onset and recurrent HF (6, 7). More than 40% of hospitalized patients with HF also have T2DM. Furthermore, T2DM leads to significant increases in the risk of hospitalization, readmission to hospital, and death among patients with HF (8). A recent meta-analysis of 129 studies involving >10 million individuals demonstrated that prediabetes is also associated significantly with a higher risk of CV diseases and all-cause death in a healthy population and in people with established atherosclerotic CV disease (9). In a recent large-scale cohort study, patients with T2DM still had a higher risk of hospitalization for HF than the control group even if the five major risk factors (hypertension, smoking, albuminuria, elevated low-density lipoprotein cholesterol, elevated glycated hemoglobin) were under control (10). Several studies have shown that patients with both T2DM and HF have a worse outcome than those with one of these diseases only (11).

Traditional anti-HF pharmacological therapies are neuroendocrine inhibitors, which modulate the renin–angiotensin–aldosterone system (RAAS) or sympathetic nervous system (SNS). Such therapies can improve QoL significantly and reduce mortality in patients with heart failure with reduced ejection fraction (HFrEF) (12). However, these drugs do not show the same significant effects in patients with heart failure with preserved ejection fraction (HFpEF) (13–15). More frustratingly, the angiotensin receptor-neprilysin inhibitor sacubitril-valsartan also did not significantly reduce the risk of hospitalization and all-cause death among patients with HFpEF (16). So far, an “ideal” drug demonstrating a convincing prognosis-improving effect in patients with HFpEF is lacking (12–16).

Trials focusing on CV outcomes have demonstrated the significant benefits of sodium-glucose co-transporter 2 (SGLT2) inhibitors in improving clinical outcomes (17–22). Based on such overwhelming evidence, SGLT2 inhibitors are being recommended increasingly to treat patients with HFrEF or T2DM at high risk of CV diseases (23–26). Moreover, some studies have suggested that they may also be beneficial for patients with HFpEF or acute HF, which merits evaluation in large-scale studies.

## Sodium-Glucose Co-Transporters (SGLTS)

Under physiological conditions, glucose is filtered from glomeruli and reabsorbed in the S1 segment of the proximal convoluted tubule. Upon exceeding the absorptive capacity, redundant glucose is excreted through urine (27). Renal glucose reabsorption is mediated mainly by SGLTs. The latter are a family of membrane proteins found chiefly in the mucosa of the small intestine and in the proximal convoluted tubule of the kidney (28). Among the 12 members of the SGLT family, SGLT1 and SGLT2 are the two most important isoforms involved in renal glucose reabsorption. Characterized as a high-capacity and low-affinity transporter, SGLT2 is distributed mainly in segments S1/S2 of renal proximal convoluted tubule and mediates about 80–90% of filtered glucose reabsorption. In contrast, SGLT1 is a low-capacity and high-affinity transporter. SGLT1 is found mainly in the brush border of the small intestine and in the S3 segment of the proximal convoluted tubule. SGLT1 is responsible for the residual 10–20% of glucose reabsorption (29).

## SGLT2 Inhibitors

As a novel class of oral antidiabetic agents, SGLT2 inhibitors are used initially to reduce the serum level of glycated hemoglobin and improve glycemic control. Surprisingly, several large-scale randomized clinical trials have demonstrated that SGLT2 inhibitors reduce CV mortality dramatically, improve CV outcomes, and reduce HF hospitalization in T2DM patients whether they have pre-existing CV disease or not (17–22). Recently, investigators from Harvard Medical School (Boston, MA, USA) have suggested that SGLT2 inhibitors should be considered as one of the basic medications in patients with HFrEF, in addition to standard therapies (23). With the advent of more clinical trials to further evaluate the benefits and safety of SGLT2 inhibitors in different subtypes of HF, they may become increasingly promising agents for patients with HF.

The benefits of SGLT2 inhibitors upon clinical outcomes are attributable to several complex mechanisms in addition to lowering the serum glucose level (though they were developed initially as antihyperglycemic agents).

### Hemodynamic Mechanisms of SGLT2 Inhibitors

A series of clinical trials have shown an obvious decrease in hospitalization due to HF shortly after beginning use of SGLT2 inhibitors, suggesting that they may improve hemodynamic status.

SGLT2 inhibitors have been reported to lower blood pressure by ~4.0/1.6 mmHg without increasing the heart rate, which suggests that the SNS is not activated (and may even be inhibited) (30). SNS activation is unfavorable in HF, and is often accompanied by a worse clinical outcome (31). Therefore, compared with agents which may lead to SNS activation, SGLT2 inhibitors may be more beneficial to patients with HF (31, 32). In addition, unlike the gradually decreasing glucose-lowering effect of SGLT2 inhibitors in patients with chronic kidney disease, the effect of these agents on lowering blood pressure is similar in patients with different renal conditions, even those with a lower estimated glomerular filtration rate (eGFR) (33). Early theories and evidences have suggested that their antihypertensive effect might derive from a volume-reduction mechanism due to diuresis and natriuresis. In addition, other effects, such as calorie loss, fat-mass decrease, and weight loss, resulting from increasing diuresis and glucosuria also contribute to lowering of blood pressure (34). Furthermore, recent studies have suggested that the ketogenic properties of SGLT2 inhibitors may be a reasonable explanation for their antihypertensive effects independent of renal function (35). However, the blood-pressure reduction observed with SGLT2 inhibitors cannot explain satisfactorily the rapid and effective improvement of clinical outcomes given that even those antihypertensive drugs do not demonstrate such impressive effects.

Under physiological conditions in proximal convoluted tubule, glucose and sodium ions (Na^+^) are reabsorbed together from the glomerular filtrate. By inhibiting Na^+^ reabsorption in proximal tubule, SGLT2 inhibitors promote natriuresis and reduce extracellular fluid and plasma volume. After taking SGLT2 inhibitors, the early manifestation is an increased urine volume in the first few days, then the urine volume returns gradually to a baseline level in several weeks, and a reduction of plasma volume of ~7.3% is observed after 12 weeks (36). Interestingly, compared with loop diuretics, SGLT2 inhibitors tend to remove more fluid from the interstitial space than from the circulation, resulting in more electrolyte-free water clearance (37). In consideration of an increased volume load in HF, with inadequate arterial perfusion due to impaired cardiac function, the reduction of interstitial volume may be more favorable to patients with HF. In this sense, natriuresis and reduction in volume load are likely to explain (at least in part) the protective effects of SGLT2 inhibitors in HF (22).

Arterial stiffness is recognized as an important predictive factor of the morbidity and mortality associated with HF (38) because it can lead to an increased cardiac load and further deterioration of heart function. Some cardioprotective agents, such as RAAS inhibitors, have been demonstrated to ameliorate arterial stiffness, reduce cardiac load, and improve CV outcomes. Endothelial function also plays an important part in maintaining myocardial function, hemodynamics, the systemic circulation and pulmonary circulation. Endothelial dysfunction can lead to disturbed production and utilization of nitric oxide (NO), which increases vascular resistance further (39). Pulse wave velocity (PWV) is the major parameter of arterial stiffness (40). In an observational study, dapagliflozin significantly reduced the aortic PWV (41). Similarly, in subsequent randomized controlled trials (RCTs), PWV was also decreased by empagliflozin or canagliflozin compared to placebo (42, 43). These results suggested that SGLT2 inhibitors can alleviate arterial stiffness. In addition, the improvement of endothelial function was also observed by using SGLT2 inhibitors in these studies. These beneficial effects may be mediated by increasing NO production, reducing oxidative stress, or activating voltage-gated potassium ion (K^+^) channels and protein kinase G (41, 44, 45). However, unlike the three well-recognized SGLT2 inhibitors (empagliflozin, dapagliflozin, canagliflozin), luseogliflozin did not show a similar beneficial effect on arterial stiffness in LUSCAR study (46). This may indicate that the improvement of arterial stiffness with SGLT2 inhibitors is due to specific drug rather than class effect.

Sodium–hydrogen exchangers (NHEs) transfer Na^+^ into the cell in exchange for proton export. NHEs are involved in maintenance of Na^+^ homeostasis and physiological pH (47). NHE1 is expressed in cardiomyocytes and its activation may lead to increased intracellular Na^+^ and calcium ions (Ca^2+^) (48). This effect is involved in abnormal myocardial hypertrophy and ischemia–reperfusion injury (49). NHE3 is expressed in proximal tubule and mediates tubular reuptake of Na^+^. Upregulated expression of NHE1 and NHE3 can be observed in a failing heart (50). Despite absent expression of SGLT2 in the heart, SGLT2 inhibitors can reduce intracellular concentrations of Na^+^ and Ca^2+^ and protect the heart from intracellular Ca^2+^ overload by inhibiting activation of NHE1 receptors (51). Similarly, SGLT2 inhibitors can also block NHE3, which further enhance natriuresis, restores whole-body sodium homeostasis, and improves cardiac function (52). Therefore, inhibition of NHE1 and NHE3 contributes to the cardioprotective effects of SGLT2 inhibitors.

Studies have demonstrated a slight increase in the hematocrit upon initial treatment with SGLT2 inhibitors (36). Changes in the hematocrit and hemoglobin concentration have been thought to be associated with decreased plasma volume and increased erythropoietin production. However, in the RED-HF trial, an increase in the hematocrit by erythropoietin injection showed no association with prognostic improvement, indicating that this effect could be attributed to reduction in plasma volume rather than increasing the circulating concentration of erythropoietin (53).

### Cardio–Renal Protection of SGLT2 Inhibitors

Cardio–renal interactions are critical for the occurrence and progression of HF. Impaired heart function leads to inadequate perfusion and subsequent decreased renal function. Renal dysfunction furthers exacerbate HF deterioration, and is often accompanied by a worse prognosis (54).

In T2DM patients, the initial use of SGLT2 inhibitors may induce an acute, mild, dose-dependent reduction of the eGFR over the 1st weeks. Then, the eGFR returns toward baseline gradually and remains stable over a long time (55, 56). This early decline in the eGFR may be associated with vasoconstriction of afferent arterioles in glomeruli resulting from increased tubule–glomeruli feedback (57). In hyperglycemia condition, due to upregulation of SGLTs in the kidney, renal Na^+^ reabsorption increases dramatically. In addition, activated NHE3 causes increased SGLT2 expression and leads to oxidative stress, acidosis, RAAS activation, SNS activation, and high reabsorption of Na^+^. This alteration causes a remarkable decrease in Na^+^ delivery to the macula densa, which may be misperceived as a decrease in the circulating volume, thereby leading to inappropriate afferent arteriolar vasoconstriction and subsequent increase of intraglomerular pressure and hyperfiltration. Increased intraglomerular pressure and hyperfiltration are involved in nephropathy progression.

Therapy using SGLT2 inhibitors can hinder the reabsorption of glucose and Na^+^ in proximal convoluted tubule, and may increase Na^+^ secretion by the S3 segment of proximal tubule. As a result, the Na^+^ concentration transferred to the distal macula densa increases. Thus, by regulating tubule–glomerular feedback, SGLT2 inhibitors improve glomerular afferent arteriolar adaption, alleviate abnormal intraglomerular hypertension and hyperfiltration, and achieve protection of renal function (58). This renal-protective mechanism is different from that elicited by RAAS inhibitors, which reduce intraglomerular pressure and alleviate hyperfiltration by efferent arteriolar vasodilatation. Therefore, combined use of these two agents may be more beneficial for the protection of cardio–renal function. This effect has been demonstrated in clinical and experimental studies (59, 60).

In a meta-analysis involving 48 randomized controlled trials, compared with placebo or other antidiabetic drugs, SGLT2 inhibitors exerted renal protection by decreasing albuminuria, slowing the progression from microalbuminuria to macroalbuminuria, and reducing the risk of end-stage renal disease (61).

Because of the close interaction between HF and renal dysfunction, use of SGLT2 inhibitors (especially in combination with RAAS inhibitors) can break this “vicious cycle” and provide cardio–renal protection.

### Inflammatory and Metabolic Mechanisms of SGLT2 Inhibitors

#### Inflammation

Chronic systemic inflammation plays an important part in the development of cancer, chronic kidney diseases, CV diseases, DM, and non-alcoholic fatty liver disease (62). In several clinical trials, an increased level of C reactive protein (CRP) has been observed in patients with HF (acute and chronic) (63, 64), which suggests that inflammation is prevalent in HF. In fact, systemic inflammation has become a crucial pathophysiological feature of HF, and it is thought to be closely related to the occurrence, progression, and severity of HF (65). Inflammation contributes to HF development and regulation of heart function (66). These effects are achieved through a series of signaling pathways, and numerous proinflammatory cytokines, immune-response mediators, and inflammasomes are involved (67, 68).

To be specific, endothelial inflammation can cause a decline in NO production and increased generation of reactive oxygen species (ROS) (69). Subsequently, impaired metabolism of NO can cause an obvious reduction in activity of protein kinase G. This action leads to inhibition of phosphorylation in cytoskeletal proteins, and eventually results in increased myocardial stiffness and abnormal myocardial hypertrophy (69). In addition, endothelial inflammation can upregulate expression of intercellular adhesion molecules, thereby leading to collagen deposition, microvascular dysfunction, and myocardial fibrosis (69). These adverse effects result in deterioration of heart function (especially myocardial diastolic function).

Activation of the immune system is also involved in the inflammatory process in HF. As a toll-like receptor (TLR) expressed predominantly in the heart, TLR4 is closely associated with myocardial inflammation. Upon binding with its specific ligands, TLR4 can trigger a series of signaling responses, including expression of related proinflammatory cytokines such as tumor necrosis factor (TNF)-α, interleukin (IL)-6, IL-1, and activation of inflammasomes (70). Although possibly beneficial in the short term to patients with HF, activation of the immune system is very detrimental in the long term due to the subsequent pathological cardiac remodeling and progressive deterioration of heart function. In addition, inflammatory responses affect the functions of other tissues and organs (e.g., skeletal muscles, lungs, kidneys) and lead to hypoxia, increased pulmonary arterial pressure, as well as retention of water and Na^+^, which have adverse effects on patients with HF. In view of the important role of inflammation in HF, anti-inflammatory therapy has become desirable against HF. However, in clinical trials, most of the direct anti-inflammatory or anti-cytokine therapies have not demonstrated significant benefits for improving outcomes in patients with HF (71, 72).

Several experimental studies have shown that SGLT2 inhibitors decreased the concentrations of inflammatory factors such as CRP, TNF-α, and IL-6 (73). Subsequent clinical trials confirmed the anti-inflammatory effects of SGLT2 inhibitors (74). Recently, a meta-analysis involving 23 heterogeneously designed clinical trials showed that therapy using SGLT2 inhibitors could reduce the level of CRP, IL-6, TNF-α significantly, and increase the adiponectin level significantly (75). Those results suggested that SGLT2 inhibitors can suppress inflammation, but the exact mechanisms remain unclear. Experimental studies have indicated that SGLT2 inhibitors may exert their anti-inflammatory effect through different signaling pathways, such as the nuclear factor-kappa B (NF-κB) signaling pathway (76). Other signaling pathways, including the mitogen-activated protein kinase (MAPK) pathway and TLR4 pathway, may also be involved in the anti-inflammatory effect of SGLT2 inhibitors. These effects need to be determined in further studies.

#### Oxidative Stress

Oxidative stress is another common important mechanism involved in the pathophysiology of CV disease. Oxidative stress is characterized by excessive generation of ROS and insufficient endogenous antioxidants (77). In health, a small quantity of ROS is produced by mitochondria, including xanthine oxidase, nitric oxide synthase, and the reduced form of nicotinamide adenine dinucleotide phosphate (NADPH) oxidase, which maintain a relative balance with endogenous antioxidants. However, under pathological conditions, ROS generation increases significantly due to mitochondrial dysfunction, enhanced activity of NADPH oxidase, xanthine oxidase, and nitric oxide synthase, while the endogenous antioxidants are in serious deficiency (78). This shift causes an obvious accumulation of ROS. Then, an overabundance of ROS leads to DNA damage, protein peroxidation, cellular-microenvironment disorders, subsequent cellular dysfunction, and even cell death. In the failing heart, excessive ROS leads to an increased intracellular Ca^2+^ concentration by affecting the function of Na^+^/Ca^2+^ exchangers and L-type Ca^2+^ channels, resulting in further myocardial electrophysiological abnormalities. Increased ROS also leads to enhanced activity of ryanodine receptor-2 and inhibited activity of Ca^2+^-adenosine triphosphatase-2 in sarcoplasmic reticuli, thereby resulting in Ca^2+^ overload and reduced sensitivity to Ca^2+^. In addition, accumulated ROS cause mitochondrial dysfunction, inefficiency of energy metabolism, and increased myocardial fibrosis. These seriously detrimental effects lead eventually to cardiac remodeling, contractile and diastolic dysfunction, and progressive HF (79). Some experimental studies have suggested that anti-oxidative stress therapy can improve heart function and reduce myocardial fibrosis. Unfortunately, in a series of clinical trials, therapy targeting oxidative stress did not yield encouraging results in patients with HF (80, 81).

Experimental studies have shown that SGLT2 inhibitors have the potential for antioxidative stress because they can lower free-radical production and increase expression of antioxidants such as manganese superoxide dismutase and catalase (82). In addition, because of the close relationship between hyperglycemia and oxidative stress, the antihyperglycemic effects of SGLT2 inhibitors are thought to be an important mechanism of antioxidative stress. Moreover, SGLT2 inhibitors may also exert antioxidative effects by ameliorating RAAS activity, reducing expression of proinflammatory cytokines, and improving mitochondrial function (83, 84). In recent clinical studies, SGLT2 inhibitors reduced levels of 8-iso-prostaglandin-F2α and 8-hydroxy-2′-deoxyguanosine, which are considered to be biomarkers of oxidative stress (75). That result provides evidence to the antioxidative-stress effect of SGLT2 inhibitors in patients with T2DM. Similarly, this effect may also contribute (at least in part) to the CV benefits of SGLT2 inhibitors.

#### Insulin Resistance

Insulin resistance is a prevalent pathological mechanism hidden under a series of metabolic diseases represented by T2DM. The close relationship between insulin resistance and HF has been recognized (85). In a considerable proportion of patients with HF, insulin resistance coexists and further exacerbates the deterioration of HF. In turn, HF or MI may also increase the risk of insulin resistance or T2DM significantly. Insulin exerts its biological effects through two signaling pathways: phosphatidylinositol-3 kinase/protein kinase B (PI3K/Akt) and MAPK (86).

In health, when bound to insulin, the insulin receptor auto-phosphorylates. Then, the activated insulin receptor phosphorylates insulin receptor substrate (IRS) proteins. Phosphorylated IRS binds to PI3K and activates it, which further promotes activation of its downstream signaling molecules and, ultimately, leads to Akt phosphorylation. As a result, glucose transporter (GLUT)-4 translocates to the cell membrane and mediates glucose uptake into cardiomyocytes and skeletal muscle cells. When phosphorylated IRS binds to growth factor receptor bound protein-2, the MAPK signaling pathway is activated, which is associated with myocardial hypertrophy, fibrosis, and endothelial dysfunction. In insulin resistance, oxidative stress often coexists and leads to impaired function of Na^+^/Ca^2+^ exchangers and L-type Ca^2+^ channels, resulting in impaired uptake of Ca^2+^. Then, a Ca^2+^-regulation disorder impairs cardiac contractile and diastolic function. In addition, resistance to cardiac insulin reduces the activity of the PI3K/Akt signaling pathway, which also contributes to the abnormality in Ca^2+^ uptake. In endothelial cells, insulin resistance decreases the production of NO and increases release of endothelin-1, contributing to myocardial hypertrophy, fibrosis, and apoptosis. Insulin resistance and oxidative stress also cause dysfunction in mitochondria and endoplasmic reticuli, which leads to myocardial apoptosis and metabolic insufficiency (87). These detrimental effects of insulin resistance promote the progression and deterioration of HF together.

SGLT2 inhibitors have various beneficial effects upon insulin resistance. Their antihyperglycemic effects are caused by increased urinary glucose excretion and are independent of the insulin level, so SGLT2 inhibitors can reduce excessive insulin secretion due to renal glucose absorption. This process can decrease lipogenesis and lipolysis, which leads to a reduction of fat mass and bodyweight. It can also increase glucose uptake and ketone production, which improves metabolic efficiency. In addition, SGLT2 inhibitors have been reported to reduce hyperglycemic toxicity and protect pancreatic function (88, 89). Moreover, several studies have shown that SGLT2 inhibitors can prevent mitochondrial dysfunction and improve cell viability (90). These effects of SGLT2 inhibitors contribute to an increase in insulin sensitivity and reduction of insulin resistance.

#### Energy Metabolism

Under physiological conditions, ~60–90% of cardiac energy is derived from oxidation of fatty acids. Carbohydrate is responsible for the residual 10–40% of adenosine triphosphate (ATP) production. However, in patients with HF, oxidation of fatty acids may be reduced due to insulin resistance and oxidative stress. As a result, glucose utilization increases. Due to insufficient oxygen supply, the route of glucose metabolism shifts to anaerobic glycolysis accompanied by a small amount of ATP production. As a result, in a failing heart, impaired oxidation of fatty acids and a shift in glucose-metabolism pathways lead to a significant reduction in cardiac metabolic efficiency with inadequate ATP production (91, 92).

SGLT2 inhibitors have several beneficial effects on energy metabolism in patients with HF. As mentioned above, SGLT2 inhibitors can ameliorate oxidative stress and insulin resistance, thereby reducing their detrimental effects on energy metabolism. In diet-induced obese mice, empagliflozin showed to promote fat utilization by upregulating the expression of genes related to fatty acid oxidation, and also attenuate inflammation and insulin resistance (93). In another high-fat diet mice model, canagliflozin suppressed lipid synthesis and reduced body weight. In addition, recent studies suggested that SGLT2 inhibitors can not only regulate glucose metabolism and enhance glucose tolerance by improving insulin resistance (94), but also improve the function of pancreatic beta cells (95). These effects are beneficial to regulate glucose oxidation and increase energy metabolic efficiency. Besides, as an alternative energy source, the utilization capacity of ketones depends mainly on the levels of succinyl-CoA:3-ketoacid CoA transferase (SCOT) in different tissues. In this process, β-hydroxybutyrate is converted to acetoacetate, which is oxidized further to acetoacetyl-CoA by SCOT. Then, in mitochondria, acetoacetyl-CoA is converted to acetyl-CoA, which subsequently enters the tricarboxylic-acid cycle to produce ATP. In health, ketone metabolism contributes a very small proportion of energy production. However, in a failing heart, ketones may become an important and preferred energy source due to the significant increase of SCOT when treated with SGLT2 inhibitors. Moreover, ATP is not consumed in ketone metabolism, so this process may increase metabolic efficiency and ATP production. In addition, SGLT2 inhibitors can improve mitochondrial function. These mechanisms may explain the metabolic benefits of SGLT2 inhibitors in patients with HF (96, 97).

#### Advanced Glycation End Products

Advanced glycation end products (AGEs) have been shown to be involved in diabetic nephropathy and HF. AGEs can increase arterial stiffness by interacting directly with the extracellular matrix (98). In addition, by interacting with the receptor for advanced glycation end products (RAGEs), AGEs can induce the inflammatory response, immunoreaction, oxidative stress, and fibrosis (99, 100). In a unicentric observational clinical study, Paradela-Dobarro et al. suggested that activation of the AGE-RAGE signaling pathway may contribute to HF development, and that soluble RAGEs may be critical predictors of adverse long-term outcome in HF (101). Some experimental studies have indicated reduced production of AGEs and suppression of the AGE-RAGE signaling pathway in mice after treatment with SGLT2 inhibitors (83, 102, 103). However, whether there is a similar effect on the AGE-RAGE signaling pathway using SGLT2 inhibitors in patients with HF must be studied further.

#### Obesity

Obesity is defined as a body mass index (BMI) >30 kg/m^2^. Obesity has become an increasingly prevalent social problem in developed and developing countries. It is widely recognized that obesity (especially morbid obesity) can result in an increased risk of CV complications such as hypertension, arterial atherosclerosis, ventricular hypertrophy, and HF (104, 105). However, in several clinical studies, researchers were surprised to observe that in patients with CV diseases, those who were obese tended to have better clinical outcomes and survival compared with those in a leaner group. This is known as the “obesity paradox” (106). Although the exact mechanisms of the obesity paradox are incompletely understood, most investigators have recognized that it may be inappropriate to use BMI exclusively as the evaluation factor for obesity. Subsequent studies have suggested that adjusted cardiorespiratory fitness may be a more favorable indicator to evaluate the CV risk in an obese population. In general, fat distribution may be more closely associated with HF, and abdominal adiposity has been thought to be an important predictive factor of HF (107).

Several mechanisms may be involved in the close relationship between obesity and HF. First, compared with a non-obese population, obese people have a higher tendency of ventricular hypertrophy, which results in impaired adaptive regulation of cardiac output (even if the ejection fraction appears to be in the normal range). In addition, accumulation of epicardial fat in obese patients may lead to a further decline in left-ventricular compliance and exacerbate diastolic dysfunction. Similarly, adaption of their respiratory function also decreases. Second, higher levels of proinflammatory factors, including CRP, IL-6 and TNF-α, have been observed in obese patients compared with their non-obese counterparts, suggesting that obesity tends to induce inflammation. Obesity-related inflammation can cause oxidative stress, microvascular injury, and myocardial fibrosis. In addition, obese patients have a greater plasma volume load, which may increase the excess burden to the heart and kidneys, especially if pre-existing cardio–renal decline is present. Third, the RAAS activation and inflammation induced by obesity may lead to reduced vascular compliance and volume redistribution, which may play an important part in CV congestion and cardiac dysfunction.

Several oral antidiabetics agents and insulin may lead to weight gain, which is unfavorable for patients with HF. In contrast, SGLT2 inhibitors show a weight-loss effect owing to the increased excretion of glucose (108). This effect is observed from the 1st weeks of therapy, and the bodyweight reduces further in subsequent months and is then maintained. The reduction of bodyweight results primarily from fat loss, with a significant decrease in visceral and subcutaneous adipose tissue (109). The leptin–aldosterone–neprilysin axis is thought to have a distinct role in HF patients suffering from obesity (110). SGLT2 inhibitors have been observed to alleviate the detrimental effects of leptin. As mentioned above, SGLT2 inhibitors suppress RASS activity, ameliorate systemic inflammation, and improve insulin sensitivity. In addition, these agents are also thought to diminish fat accumulation and adipokine generation. These mechanisms help to prevent the structure and function of the heart from the harmful effects induced by obesity.

Epicardial adipose tissue (EAT) is a subtype of white adipose tissue. EAT is distributed around the heart. During recent decades, the relationship between EAT and CV diseases has attracted considerable attention. Recently, EAT has been recognized as a special type of fat with peculiar anatomic, biomolecular, and genetic features. In health, EAT appears as an “organ” for storing free fatty acids. However, overabundant free fatty acids may lead to excessive production of ROS and alteration of the intracellular Ca^2+^ concentration, which are components of diastolic dysfunction. EAT can also induce insulin resistance and inflammatory reactions, which are closely associated with a series of CV diseases (especially HF). Moreover, EAT is a crucial secretory organ. It can release a range of adipokines (IL-6, IL-1β, TNF-α) that can cause myocardial lipo-toxicity and impair heart function further. EAT can also secrete adiponectin (a cardioprotective adipocytokine), which achieves beneficial effects by reducing the inflammatory response and apoptotic activity. In addition, EAT has been shown to secrete various microRNAs, which are necessary in the formation and development of the heart. Numerous microRNAs demonstrate different effects on regulating the metabolism of glucose and lipids, myocardial hypertrophy, and cardiac fibrosis. Due to its metabolic and secretory activity, EAT is thought to have a critical role in the pathophysiological process of several CV diseases, including coronary heart disease, cardiomyopathy, and HF (111).

Experimental studies have indicated that use of SGLT2 inhibitors is associated with a significant increase in the adiponectin concentration, inhibited activities of inflammatory cells, and suppressed expression of proinflammatory cytokines. In a small clinical study, treatment with SGLT2 inhibitors reduced the EAT volume and decreased the TNF-α level. Several other studies have also demonstrated that SGLT2 inhibitors reduce the accumulation and alter the distribution of epicardial fat (112–114). Those data suggest that SGLT2 inhibitors exert their benefits (at least in part) in patients with HF by regulation and distribution of EAT.

#### Hyperuricemia

Hyperuricemia is prevalent in patients with HF or T2DM. The serum level of uric acid is an important predictor of a poor prognosis in HF. An increased level of uric acid is thought to be associated with RAAS activation, oxidative stress, systemic inflammation, and endothelial dysfunction. On the one hand, the products of purine degradation increase significantly due to increased glycolysis and insufficient production of ATP during hypoxemia. On the other hand, reduced cardiac output and inadequate renal perfusion cause RAAS activation, which leads to increased reabsorption and decreased excretion of uric acid. Conventional diuretic therapy often causes an increased level of uric acid, which is unfavorable for patients with HF (115, 116). Differently, SGLT2 inhibitors have been shown to reduce the serum level of uric acid and contribute to reduce the CV risk (117, 118). As mentioned above, SGLT2 inhibitors can improve energy metabolism and increase ATP generation, which are beneficial to reduce purine metabolites. Furthermore, because of increased excretion of glucose in urine, the functions of GLUT-9 and uric acid transporter-1 (expressed in renal tubular epithelial cells) are inhibited. Thus, suppressed reabsorption and enhanced excretion of uric acid finally result in the reduction of the serum level of uric acid.

In brief, SGLT2 inhibitors exert CV-protective effects through various complex mechanisms, which merit further study.

#### SGLT2 Inhibitors in HFpEF

As a special phenotype of HF, HFpEF has received increasing attention due to its rapidly rising incidence and numerous complications. Compared with HFrEF, HFpEF is a more complicated clinical syndrome with multiple etiologies and unclear pathogenesis. HFpEF accounts for more than half of hospitalized patients with HF. First, the major risk factors of HFpEF are more diverse and complicated. In addition to coronary heart disease, hypertension, T2DM, and obesity, other factors are involved in the pathological process of HFpEF: anemia, chronic obstructive pulmonary disease, inflammation, and being female. Second, in patients with HFrEF, the manifestation of cardiac remodeling is enlarged ventricular volume and reduced ejection fraction. In contrast, cardiac remodeling is usually characterized by myocardial hypertrophy, decreased cardiac compliance, and subsequent diastolic dysfunction. Third, in HFrEF patients, inflammation may be a consequence of HF only, whereas inflammation is thought to be a crucial basis of HFpEF and closely associated with myocardial stiffness, fibrosis, and impaired diastolic function mediated by multiple cellular signaling pathways (119). In addition, RAAS activation and EAT are also involved in the occurrence and development of HFpEF. Moreover, unlike the obvious advances in drug and device therapies in HFrEF, treatments for HFpEF are more difficult and challenging. Current treatments for HFpEF are limited to alleviating symptoms and controlling the primary disease. To date, no convincing treatment, not even RAAS or SNS inhibitors, has been shown to reduce morbidity or mortality significantly in patients with HFpEF (120).

In view of the broad biological effects, including lowering of serum levels of glucose and blood pressure, reducing plasma volume, suppressing inflammation and oxidative stress, improving insulin resistance and increasing energy metabolism, SGLT2 inhibitors can maintain cardiac hemodynamic and electrophysiological stability, alleviate abnormal hypertrophy and remodeling, and improve myocardial systolic and diastolic function which are, theoretically, beneficial to patients with HFpEF.

In a female diabetic rodent model, empagliflozin showed to improve diastolic function and reduce fibrosis (121). Similarly, in a non-diabetic rodent model of HFpEF, empagliflozin improved cardiac diastolic function and reduced wall stress. The potential pathophysiological mechanisms included reduced preload and altered hemodynamics (122). In a multi-hit mouse model of HFpEF, dapagliflozin also improved cardiac function and tissue fibrosis (123). Tofogliflozin, another SGLT2 inhibitor, ameliorated cardiac hypertrophy and fibrosis in dahl salt-sensitive and salt-resistant rats fed a high-fat diet (124). These experimental studies suggest that SGLT2 inhibitors may be beneficial for patients with HFpEF. HFpEF is a more prevalent phenotype of HF in T2DM patients. In large-scale clinical studies such as EMPA-REG OUTCOME, the consistent and significant benefits of different SGLT2 inhibitors for T2DM patients carrying a high CV risk suggest the potential therapeutic value of these agents for HFpEF. However, in a single-center, retrospective study, SGLT2 inhibitors did not appear to improve left ventricular reverse remodeling in patients with T2DM and HFpEF (125). But, considering the relatively short observation time, this study is not enough to suspect the effect of SGLT2 inhibitors for HFpEF. In the MUSCAT-HF study, although BNP concentration decreased after treatment of luseogliflozin or voglibose, no significant difference in the degree of reduction in BNP was observed between these two groups (126). But, in this study, the most of patients enrolled were at low risk, with a low degree of HF and a low baseline level of BNP. In addition, about 40% patients did not have pre-existing atherosclerotic CV disease. Since SGLT2 inhibitors may be more effective in patients at high CV risk, these biases may lead to an underestimated effect of SGLT2 inhibitors for HFpEF (126). Until now, whether patients with HFpEF will benefit from SGLT2 inhibitors remains controversial. The ongoing EMPEROR-Preserved trial will assess the effects of empagliflozin on morbidity and mortality in patients with HFpEF. In addition, other several studies such as DELIVER and PRESERVED-HF are also underway to evaluate the benefit of SGLT2 inhibitors in patients with HFpEF. If the expected result is achieved, SGLT2 inhibitors may prove to be efficacious treatment for patients with HFpEF (127).

#### SGLT2 Inhibitors in Acute HF

Acute HF is characterized by the sudden occurrence or rapidly worsening symptoms and/or signs of HF. As an extremely dangerous and potentially life-threatening clinical condition, acute HF requires urgent diagnosis and treatment. Acute decompensated HF, which is frequently induced by acute myocardial ischemia, severe infection, severe arrhythmia, uncontrolled hypertension, or severe disorders in the internal environment, tends to be associated with high mortality and expensive healthcare costs during and after hospitalization (128). Diuretics and vasodilators can relieve the clinical symptoms of acute HF, but pharmacological agents known to improve the prognosis significantly in patients with acute HF are lacking.

Given that they increase natriuresis, reduce cardiac load, improve renal function, and have excellent performance in treatment of chronic HF, SGLT2 inhibitors are hypothesized to be beneficial in acute HF as well. A *post-hoc* analysis of the EMPA-REG OUTCOME trial showed that empagliflozin may reduce risk of the post-acute HF rehospitalization and mortality, which provide a cogent rationale for SGLT2 inhibitors in patients hospitalized with decompensated HF (129). In a single-center prospective study of acute HF, dapagliflozin was associated with decreased all-cause mortality and readmission despite the small sample size (130). In the EMPA-RESPNSE-AHF trial (the first study to evaluate the effects and safety of SGLT2 inhibitors in acute decompensated HF), despite the small sample size, these agents reduced the risk of rehospitalization for HF or death within 60 days, *vs*. placebo (131). In a recent retrospective analysis of a series of patients with acute decompensated HF, the researchers found that SGLT2 inhibitors improved weight loss, urine output, and diuretic efficiency without adverse change in renal function, blood pressure or electrolytes when used in combination with loop diuretics (132). This may partly explain the benefit of SGLT2 inhibitors in patients with acute HF, although the exact mechanism of action of SGLT2 inhibitors in acute HF is not known. However, due to the limitations of sample size or study method, it is not completely determined whether SGLT2 inhibitors can be safely and effectively applied in patients with acute HF. Large-scale randomized clinical trials are required to further evaluate the possible beneficial role of SGLT2 inhibitors in patients with acute HF (133).

#### Differences Between SGLT2 Inhibitors

Since phlorizin was first isolated from the root of an apple tree in 1,835, more than a dozen SGLT inhibitors have been developed (134). Due to the significant reduction of CV risk with empagliflozin, dapagliflozin, and canagliflozin in patients with T2DM, these benefits were once considered as class effects of SGLT2 inhibitors (135). However, recent studies showed inconsistent results, suggesting that SGLT2 inhibitors may have heterogeneity (136).

The differences in selectivity of SGLT2 inhibitors for SGLT2 vs. SGLT1 are summarized in Table 1 (134, 137, 138), which may be the main factor leading to their different pharmacological profiles and clinical effects. For example, several SGLT2 inhibitors such as empagliflozin, dapagliflozin, canagliflozin, tofogliflozin showed to alleviate arterial stiffness (41–43, 139), while luseogliflozin and ipragliflozin did not demostrate the similar beneficial effect (46, 140). In previous studies, empagliflozin, dapagliflozin, canagliflozin, luseogliflozin, and ipragliflozin reduced the biomarkers of inflammation and oxidative stress (73–75, 112, 114), however, whether ertugliflozin and sotagliflozin had the similar effect has not been reported.

**Table 1:** Differences between SGLT inhibitors in selectivity for SGLT2 vs. SGLT1.

**SGLT inhibitor**	**IC_**50**_ for SGLT1 (nM)**	**IC_**50**_ for SGLT2 (nM)**	**Selectivity (SGLT2 vs. SGLT1)**
Empagliflozin	8,300	3.1	~2,680
Dapagliflozin	1,400	1.2	~1,200
Canagliflozin	710	2.7	~260
Ertugliflozin	1,960	0.87	~2,250
Sotagliflozin	36	1.8	~20
Tofogliflozin	12,000	6.4	~1,875
Luseogliflozin	3,990	2.3	~1,730
Ipragliflozin	3,000	5.3	~560
Phlorizin	290	21	~10

*IC_50_ inhibitor concentration at half-maximal response*.

In four large-scale clinical trials, although SGLT2 inhibitors showed significant benefits in HF hospitalization (HHF), other outcomes including MACE were inconsistent. The results are summarized in Table 2 (17, 19, 21, 141). In newly published SCORED trial, sotagliflozin showed to reduce the risk of HHF and urgent visits for HF compared to placebo, however there was no significant difference in CV death between the two groups (142).

**Table 2:** Cardiorenal outcomes of SGLT2 inhibitors.

**SGLT2 inhibitor (clinical trial)**	**MACE HR (95% CI)**	**CV death HR (95% CI)**	**HHF HR (95% CI)**	**Composite renal outcomes HR (95% CI)**
Empagliflozin (EMPA-REG OUTCOME)	0.86^**^ (0.74–0.99)	0.62^**^ (0.49–0.77)	0.65^**^ (0.50–0.85)	0.54^**^ (0.40–0.75)
Dapagliflozin (DECLARE-TIMI 58)	0.93 (0.84–1.03)	0.98 (0.82–1.17)	0.73^**^ (0.61–0.88)	0.53^**^ (0.43–0.66)
Canagliflozin (CANNAS program)	0.86^**^ (0.75–0.97)	0.87 (0.72–1.06)	0.67^**^ (0.52–0.87)	0.60^**^ (0.47–0.77)
Ertugliflozin (VERTIS CV)	0.97 (0.85–1.11)	0.92 (0.77–1.11)	0.70^**^ (0.54–0.90)	0.81 (0.63–1.04)

*HR, hazard ratio; CI, confidence interval*;

** *statistical significance*.

Differences in chemical structure and pharmacological characteristics may partly explain the inconsistent results of clinical trials. However, the detailed heterogeneity among SGLT2 inhibitors needs to be further studied.

## Conclusions

Due to a series of favorable hemodynamic and metabolic effects, SGLT2 inhibitors have been transformed gradually into an important class of anti-HF drugs instead of just a novel group of antidiabetic agents. Increasingly, SGLT2 inhibitors are being recommended to improve clinical outcomes in patients with HFrEF or T2DM carrying a high CV risk. Researchers from Harvard Medical School have suggested that SGLT2 inhibitors should also be used as a new cornerstone drug for HFrEF, in addition to RAAS inhibitors, β-receptor blockers, and aldosterone antagonists. On this basis, in consideration of their multiple beneficial mechanisms and impressive performance in HFrEF, SGLT2 inhibitors have also attracted increasing attention in the field of HFpEF and acute HF. While some small-scale studies have shown encouraging results of SGLT2 inhibitors in patients with HFpEF or acute HF, others have shown inconsistent results. Although controversial, SGLT2 inhibitors are considered as a promising treatment for patients with HFpEF or acute HF. Ongoing or upcoming clinical studies will further evaluate their safety and efficacy in patients with HFpEF or acute HF, and the results will be awaited eagerly.

## Author Contributions

All authors significantly contributed to this work and approved the final version of the manuscript.

## Conflict of Interest

The authors declare that the research was conducted in the absence of any commercial or financial relationships that could be construed as a potential conflict of interest.
